# Epidemic of Invasive Pneumococcal Disease, Western Canada, 2005–2009

**DOI:** 10.3201/eid1805.110235

**Published:** 2012-05

**Authors:** Gregory J. Tyrrell, Marguerite Lovgren, Quazi Ibrahim, Sipi Garg, Linda Chui, Tyler J. Boone, Carol Mangan, David M. Patrick, Linda Hoang, Greg B. Horsman, Paul Van Caeseele, Thomas J. Marrie

**Affiliations:** Provincial Laboratory for Public Health (Microbiology) Edmonton, Alberta, Canada (G.J. Tyrrell, M. Lovgren, Q. Ibrahim, S. Garg, L Chui, T.J. Boone, C. Mangan, T.J. Marrie);; University of Alberta, Edmonton (G.J. Tyrrell, M. Lovgren, Q. Ibrahim, S. Garg, L Chui, T.J. Boone, C. Mangan, T.J. Marrie);; British Columbia Centre for Disease Control, Vancouver, British Columbia, Canada (D.M. Patrick, L. Hoang);; Saskatchewan Disease Control Laboratory, Regina, Saskatchewan, Canada (G.B. Horsman);; Cadham Provincial Laboratory, Winnipeg, Manitoba, Canada (P. Van Caeseele)

**Keywords:** Streptococcus pneumoniae, serotype 5, epidemic, invasive pneumococcal disease, bacteria, Canada, IPD, surveillance

## Abstract

In Canada before 2005, large outbreaks of pneumococcal disease, including invasive pneumococcal disease caused by serotype 5, were rare. Since then, an epidemic of serotype 5 invasive pneumococcal disease was reported: 52 cases during 2005, 393 during 2006, 457 during 2007, 104 during 2008, and 42 during in 2009. Of these 1,048 cases, 1,043 (99.5%) occurred in the western provinces of Canada. Median patient age was 41 years, and most (659 [59.3%]) patients were male. Most frequently representing serotype 5 cases (compared with a subset of persons with non–serotype 5 cases) were persons who were of First Nations heritage or homeless. Restriction fragment-length polymorphism typing indicated that the epidemic was caused by a single clone, which multilocus sequence typing identified as sequence type 289. Large pneumococcal epidemics might go unrecognized without surveillance programs to document fluctuations in serotype prevalence.

## Abstract 2

A single clone of *Streptococcus pneumoniae* serotype 5 caused this epidemic.

Before the advent of antimicrobial drugs, outbreaks of invasive pneumococcal disease were numerous. Since then, however, outbreaks have been less frequently reported and have involved fewer persons, usually those confined to closed settings such as hospitals or military barracks (*1**,**2*). Even more rare have been large outbreaks or epidemics of invasive pneumococcal disease; if and when they do occur, they tend to be caused by a limited number of pneumococcal serotypes (*2**–**4*).

The serotype of a *Streptococcus pneumoniae* bacterium is designated according to the organism’s polysaccharide capsule, its major virulence factor. Worldwide, 91 polysaccharide capsular serotypes have been identified (*5**,**6*). A small subset of serotypes is responsible for most large outbreaks; these serotypes typically include, but are not restricted to, serotypes 1, 4, 5, 9V, 12F, and 23F (*2*).

Before 2005, large outbreaks of pneumococcal disease, including invasive pneumococcal disease caused by serotype 5, were rare in Canada. In 2002, an outbreak caused by *S. pneumoniae* in northern Quebec, Canada, was reported, and blood culture identified 10 cases as being caused by a serotype 1 strain (*7*). We report a large epidemic of invasive pneumococcal disease caused by *S. pneumoniae* serotype 5 in Canada that occurred during 2005–2009. The study received approval from the institutional research review committees of the health regions and the University of Alberta ethics review board.

## Materials and Methods

In Canada, invasive pneumococcal disease is nationally notifiable. For this study, cases of invasive pneumococcal disease were defined according to the national case definition: isolation of *S. pneumoniae* from a normally sterile site, such as blood, cerebrospinal fluid, pleural fluid, biopsy tissue, joint aspirate, pericardial fluid, or peritoneal fluid (*8*). In the provinces affected by the 2005–2009 epidemic, clinical diagnostic microbiology laboratories were required by provincial health authorities to submit isolates from patients with invasive pneumococcal infections to their respective provincial laboratories, which would then send them to the National Centre for Streptococcus, in Edmonton, Alberta, for capsular serotyping and antimicrobial drug resistance epidemiologic profiling. For this study, 1 isolate per case was counted. Multiple isolates of the same serotype collected from the same patient within a 30-day period were considered to account for 1 case. Regardless of serotype, isolates collected from the same patient >30 days after the first isolate were counted as separate cases.

### Clinical Data Collection

To elucidate features of disease caused by *S. pneumoniae* serotype 5, we reviewed all cases of invasive pneumococcal disease in the northern Alberta area reported from 2005 through 2009. During the study period, Alberta was subdivided into 9 health regions. For cases originating in health regions 4 through 9 (located in northern Alberta), an extensive medical chart review was conducted. The total population for these health regions in 2008 was 1,888,881 (www.health.alberta.ca/documents/Population-Projections-2006.pdf). The clinical data collected were patient age, sex, aboriginal status (i.e., First Nations heritage), homelessness, substance abuse, type of invasive pneumococcal disease, outcome, and concurrent conditions (Table 1).

**Table 1:** Characteristics of 1,112 patients with pneumococcal disease, northern Alberta, Canada, 2005–2009*

Characteristic	Total	*Streptococcus pneumoniae* serotype		p value
		Not serotype 5, n = 827	Serotype 5, n = 285	
Demographic				
Age, mean ± SD, y	45.4 ± 22.5	47.1 ± 23.9	40.6 ± 16.7	<0.001
Age group, y				
<16	137 (12.3)	119 (14.4)	18 (6.3)	<0.001
16–65	771 (69.3)	522 (63.1)	249 (87.4)	
>65	204 (18.4)	186 (22.5)	18 (6.3)	
Male sex	659 (59.3)	471 (57.0)	188 (66.2)	0.006
First Nations heritage	145 (13.0)	83 (10.0)	62 (21.8)	<0.001
Homeless.	85 (7.6)	39 (4.7)	46 (16.1)	<0.001
Substance abuse				
Tobacco	687 (61.8)	471 (57.0)	216 (75.8)	<0.001
Alcoholism	257 (23.1)	159 (19.2)	98 (34.4)	<0.001
Illicit drug	259 (23.3)	135 (16.3)	124 (43.5)	<0.001
Concurrent conditions				
Cancer	103 (9.3)	96 (11.6)	7 (2.5)	<0.001
<5 y before IPD	103 (9.3)	96 (11.6)	7 (2.5)	<0.001
>5 y before IPD	41 (3.7)	33 (4.0)	8 (2.8)	0.361
Central nervous system disorder†	167 (15.0)	135 (16.3)	32 (11.2)	0.038
Cardiovascular disease‡	317 (28.5)	281 (34.0)	36 (12.6)	<0.001
Hematologic abnormality§	80 (7.2)	76 (9.2)	4 (1.4)	<0.001
Diabetes mellitus	134 (12.1)	119 (14.4)	15 (5.3)	<0.001
Cirrhosis	53 (4.8)	48 (5.8)	5 (1.8)	0.006
Chronic renal failure¶	48 (4.3)	46 (5.6)	2 (0.7)	<0.001
HIV/AIDS	46 (4.1)	29 (3.5)	17 (6.0)	0.072
Rheumatoid arthritis	21 (1.9)	19 (2.3)	2 (0.7)	0.088
Systemic lupus erythematosus	9 (0.8)	8 (1.0)	1 (0.4)	0.461
Mental problem#	180 (16.2)	141 (17.0)	39 (13.7)	0.183
Musculoskeletal impairment**	201 (18.1)	172 (20.8)	29 (10.2)	<0.001
Chronic obstructive pulmonary disease	161 (14.5)	131 (15.8)	30 (10.5)	0.028
Hepatitis C	157 (14.1)	91 (11.0)	66 (23.2)	<0.001
Type of pneumococcal disease				
Bacteremia	1054 (94.8)	782 (94.6)	272 (95.4)	0.564
Pneumonia	887 (79.8)	617 (74.6)	270 (94.7)	<0.001
Meningitis	68 (6.1)	66 (8.0)	2 (0.7)	<0.001
Outcome				
Death	126 (11.3)	117 (14.1)	9 (3.2)	<0.001
Hospitalization				
No. hospitalized	826	581	245	
Length of stay, d, mean ± SD	17.9 ± 39.7	20.1 ± 46.4	12.9 ± 13.1	0.001

*All values are no. (%) unless indicated otherwise. IPD, invasive pneumococcal disease. †Chronic central nervous system leak, epilepsy or other seizure disorder, Parkinsonism or other neurodegenerative disorder, Alzheimer or other dementia, or stroke or other neurologic disease. ‡Congestive heart failure, coronary artery disease, myocardial infarction, arrhythmia, congenital defect, atrial fibrillation, hypertension, or other. §Sickle cell anemia, other anemia, bleeding disorder/coagulopathy, or other. ¶Nephritic syndrome or other. #Depression or other. **Osteoarthritis, osteoporosis, or other.

### Identification and Serotyping

As part of its serotyping program, the National Centre for Streptococcus pneumococcal surveillance confirmed isolates as *S. pneumoniae* according to morphologic appearance and optochin susceptibility (*9*). All pneumococcal isolates that exhibited a positive quellung reaction when commercial type-specific antiserum (Statens Serum Institute, Copenhagen, Denmark) was used were assigned a serotype (*10*). Strains that were susceptible to optochin but for which no serotype was assigned were further tested by using the AccuProbe *Streptococcus pneumoniae* Culture Identification Test (Gen-Probe, San Diego, CA, USA) to confirm species identification.

### Antimicrobial Drug Susceptibility Testing

Drug susceptibility was determined by using the reference broth microdilution method described by the Clinical and Laboratory Standards Institute (*11*). The following antimicrobial drugs were tested: penicillin, cefotaxime, ceftriaxone, chloramphenicol, erythromycin, clindamycin, tetracycline, trimethoprim/sulfamethoxazole, levofloxacin, and vancomycin. All antimicrobial agents were purchased from Sigma-Aldrich Canada Ltd, Oakville, Ontario, Canada. Interpretation of MICs was based on Clinical and Laboratory Standards Institute performance standards that were current at the time of testing (M100-S15 through M100-S17) (*12**,**13*).

### Pulsed-field Gel Electrophoresis and Multilocus Sequence Typing

*S. pneumoniae* chromosomal DNA was prepared as described (*14*). Chromosomal DNA was restricted with 20 U of *Sma*I (New England Biolabs, Beverly, MA, USA), and pulsed-field gel electrophoresis (PFGE) was performed by using a CHEF DR-III apparatus (Bio-Rad Laboratories [Canada] Limited, Mississauga, ON, Canada) for 23 h. The parameters used were as follows: initial pulse 3.5 s, final pulse 23.5 s, voltage 6 V/cm, and temperature 13°C. *Salmonella* Braenderup U9812 was used as a molecular size marker. The macrorestriction pattern was analyzed by using Bionumerics version 5 (Saint-Martens-Latem, Belgium).

Multilocus sequence typing was performed as described (*15*). The multilocus sequence type (MLST) was searched against the online pneumococcal database (http://spneumoniae.mlst.net).

### Statistical Analyses

Data were analyzed by using SAS software version 9.1 (SAS Institute, Inc., Cary, NC, USA). Possible factors associated with serotype 5 among patients with pneumococcal disease were assessed. We examined the association of each demographic, substance abuse, and concurrent condition variable with outcome variable serotype 5 (yes or other serotype). For continuous variables, we used the *t* test or Mann Whitney U test as appropriate. For categorical variables, we used the χ^2^ or Fisher exact test. For variables that were significant (p<0.20) on univariable analyses, we used the following multivariable logistic regression model:

LOGIT P (Serotype 5) = *B*_0_ + *B*_1_*X*_1_ + *B*_2_*X*_2_ + … + *B*_p_*X*_p_ …… (1)

In model 1, the significance of variables was assessed by using the Wald statistic. The variables that were significant at p<0.05 were retained in the model. All others were removed from the model unless they were possible confounders. In the final model, we tested βs, the effect of each variable on log odds of serotype 5 after adjustment for other associated variables. To calculate rates, we used the populations that were current for each province in January 1, 2009 (*16*).

## Results

### The Epidemic

**Figure 1:**
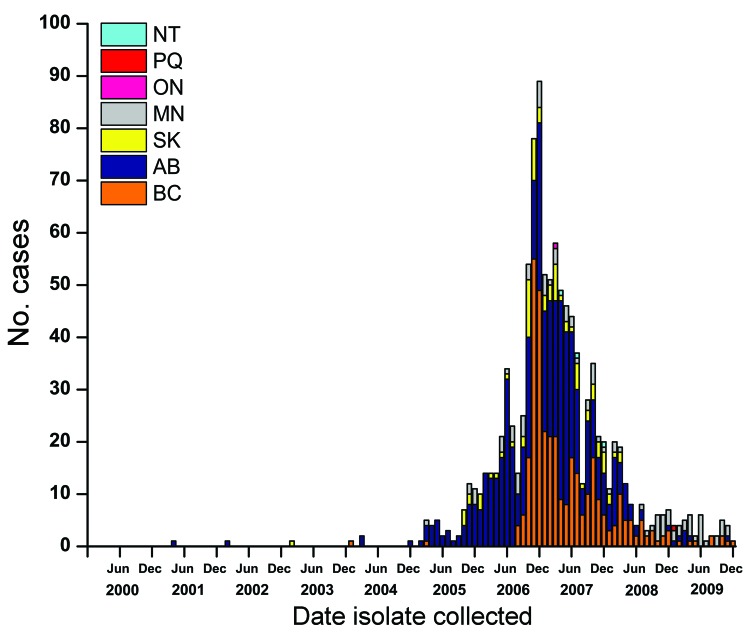
*Streptococcus pneumoniae* serotype 5 isolated in western Canada, 2000–2009, by province and month. NT, Nunavut; PQ, Quebec; ON, Ontario; MN, Manitoba; SK, Saskatchewan, AB, Alberta; BC, British Columbia.

From January 1, 2000, through December 31, 2004, the National Centre for Streptococcus serotyped 5,509 *S. pneumoniae* isolates from patients with invasive pneumococcal disease (1 isolate counted per case) from across Canada and identified 7 as serotype 5: one each in 2001, 2002, and 2003 and 4 in 2004. Since then, the Centre identified 52 isolates from patients with invasive pneumococcal disease as serotype 5 in 2005, 393 in 2006, 457 in 2007, 104 in 2008, and 42 in 2009, for a total of 1,048 cases (Figure 1). The number of cases caused by serotype 5 peaked in December 2006 and then slowly declined in 2007, 2008, and 2009 (Figure 1). Patients with invasive pneumococcal disease caused by *S. pneumoniae* serotype 5 were from British Columbia (343 [32.7%], 7.8 cases/100,000 population) Alberta (523 [49.9%], 14.4/100,000), Saskatchewan (85 [8.1%], 8.3/100,000), and Manitoba (92 [8.8%], 7.6/100,000) (Figure 1). During this 5-year period, only 5 isolates of serotype 5 were detected from elsewhere in Canada: 1 from Ontario in March 2007; 1 from Quebec in June 2009; and 3 from Northwest Territories in April, July, and December 2007.

**Figure 2:**
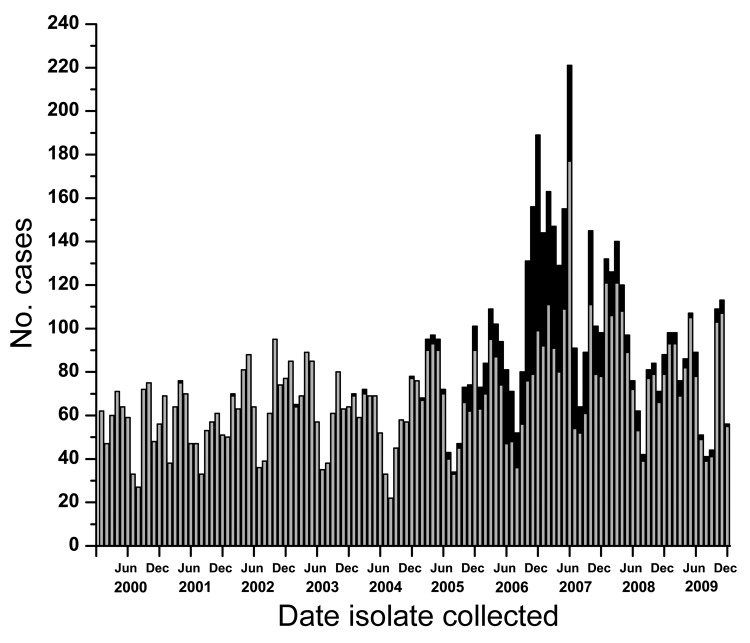
Total pneumococci serotyped in British Columbia, Alberta, Saskatchewan, and Manitoba, Canada, by month collected, 2000–2009. Gray bars represent all *Streptococcus pneumoniae* serotypes except serotype 5; black bars represent serotype 5 isolates only.

In western Canada during 2000–2009, the numbers of serotype 5 and other serotype isolates identified increased (Figure 2). The increased number of isolates submitted for typing after the onset of the epidemic indicates greater interest on the part of public health officials in western Canada in identifying circulating serotypes from patients with invasive pneumococcal disease in their provinces.

**Figure 3:**
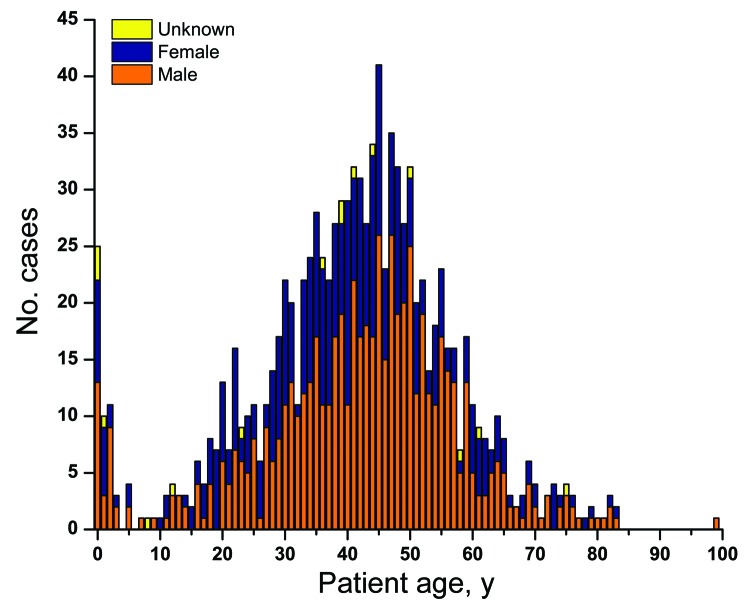
Age and sex of patients with invasive pneumococcal pneumonia caused by *Streptococcus pneumoniae* serotype 5, western Canada, 2000–2009. Median age 41 years.

The epidemic primarily affected young adults (median age 41 years) (Figure 3). Only a small subset of cases occurred among patients <5 years of age and even fewer in those >65 years of age. Most patients were male (637 male, 395 female, and 16 unknown).

### Specimen Source

The sources of specimens for the serotype 5 isolates from across Canada were as follows: 988 isolates from blood, 33 from lung/pleural fluid, 9 from cerebrospinal fluid, 7 from synovial fluid, 7 from chest/hip/leg fluid, 3 from pericardial fluid, and 1 from peritoneal fluid. For the univariable and multivariable analyses, isolates from patients with serotype 5 and nonserotype 5 invasive *S. pneumoniae* were collected from northern Alberta only (1,112 cases).

### Patient Characteristics

According to univariable analysis, serotype 5 was more prevalent than other serotypes among patients who were male (66.2% vs. 57.0%), of First Nations heritage (21.8% vs. 10.0%), or homeless (16.1% vs. 4.7%) (Table 1). Among the substance-abuse categories, associations with tobacco use, alcoholism, and illicit drug use were considered significant (p<0.001 for each; Table 1). With respect to concurrent conditions, cases of invasive pneumococcal disease caused by serotype 5 were significantly associated with cancer within 5 years before onset of invasive pneumococcal disease, cardiovascular disease, hematologic abnormalities, diabetes mellitus, cirrhosis, chronic renal failure, musculoskeletal impairment, and hepatitis C (Table 1). For patients with bacteremia and pneumonia, invasive pneumococcal disease caused by *S. pneumoniae* serotype 5 occurred significantly more often with pneumonia than did that caused by other serotypes (94.7% vs. 74.6%; Table 1). In addition, meningitis was more common for patients in the non–serotype 5 group than in the serotype 5 group (8.0% vs. 0.7%, respectively; p<0.001; Table 1). Death was less associated with infection caused by serotype 5 than by other serotypes (3.2% vs. 14.1%, respectively; Table 1).

The multivariable logistic regression model used to examine the associations of different factors with *S. pneumoniae* serotype 5 that were identified by univariable analyses found that First Nations heritage and homelessness were significantly associated with serotype 5 (adjusted odds ratio [aOR] 2.34; 95% CI 1.53–3.57 and aOR 1.83, 95% CI, 1.07–3.12, respectively) (Table 2). Tobacco use (aOR 1.90, 95% CI 1.29–2.81) and illicit drug use (aOR 1.89, 95% CI, 1.31–2.73) were also significantly associated, whereas alcoholism was not (Table 2). Among concurrent conditions, the following were significantly associated: cancer within 5 years before invasive pneumococcal disease (aOR 0.32, 95% CI 0.14–0.71), cardiovascular disease (aOR 0.51, 95% CI 0.32–0.82), hematologic abnormalities (aOR 0.19, 95% CI 0.06–0.55), and cirrhosis (aOR 0.18; 95% CI 0.06–0.50) (Table 2). Associations with musculoskeletal disease and hepatitis C infection, although significant according to univariable analysis, were not significant according to multivariable analysis.

**Table 2:** Patient characteristics associated with *Streptococcus pneumoniae* serotype 5 invasive pneumococcal disease, northern Alberta, Canada, 2005–2009*

Characteristic	Adjusted odds ratio (95% CI)	p value
Demographic		
Age group, y		
<16	0	
16–65	2.10 (1.14–3.89)	0.018
>65	1.09 (0.48–2.47)	0.846
Male sex	1.13 (0.82–1.56)	0.443
First Nations heritage	2.34 (1.53–3.57)	<0.001
Homeless	1.83 (1.07–3.12)	0.026
Substance abuse		
Tobacco	1.90 (1.29–2.81)	0.001
Alcoholism	1.19 (0.82–1.75)	0.363
Illicit drug	1.89 (1.31–2.73)	0.001
Concurrent condition		
Cancer within 5 y before IPD	0.32 (0.14–0.71)	0.005
Central nervous system disorder†	0.83 (0.52–1.33)	0.445
Cardiovascular disease‡	0.51 (0.32–0.82)	0.006
Hematologic abnormality§	0.19 (0.06–0.55)	0.002
Diabetes mellitus	0.60 (0.32–1.12)	0.109
Cirrhosis	0.18 (0.06–0.50)	0.001
Chronic renal failure¶	0.34 (0.08–1.55)	0.155
Rheumatoid arthritis	0.40 (0.09–1.84)	0.239
Mental problem#	0.64 (0.41–0.99)	0.045
Musculoskeletal impairment**	0.72 (0.44–1.18)	0.191
Chronic obstructive pulmonary disease	1.19 (0.71–2.00)	0.516

*IPD, invasive pneumococcal disease. Adjusted odds ratios were obtained by multivariable logistic regression analysis. †Chronic central nervous system leak, epilepsy or other seizure disorder, Parkinsonism or other neurodegenerative disorder, Alzheimer or other dementia, or stroke or other neurologic disease. ‡Congestive heart failure, coronary artery disease, myocardial infarction, arrhythmia, congenital defect, atrial fibrillation, hypertension, or other. §Sickle cell anemia, other anemia, bleeding disorder/coagulopathy, or other. ¶Nephritic syndrome or other. #Depression or other. **Osteoarthritis, osteoporosis, or other.

### *S. pneumoniae* Serotype 5 Characteristics

Antimicrobial drug susceptibility testing of 1,009 isolates indicated that all *S. pneumoniae* serotype 5 isolates tested were susceptible to cefotaxime, ceftriaxone, tetracycline, levofloxacin, and vancomycin. A small percentage (5 [0.5%]) of the 1,009 were intermediately resistant to penicillin (MIC ≥0.125 mg/L), 2 (0.2%) were resistant to chloramphenicol, 4 (0.4%) were resistant to clindamycin, 2 (0.2%) were intermediately resistant to erythromycin, and 4 (0.4%) were fully resistant to erythromycin. For trimethoprim/sulfamethoxazole, 976 (96.7%) isolates showed intermediate resistance (MICs 1.0–2.0 mg/L) and 18 (1.8%) showed resistance (4.0 to >16.0 mg/L). The remaining 39 isolates were not available for testing.

**Figure 4:**
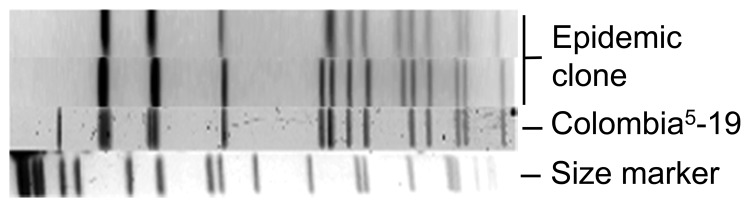
Restriction fragment length polymorphism pattern of *Streptococcus pneumoniae* serotype 5 from epidemic in western Canada, 2000–2009 (epidemic clone), determined by pulsed-field gel electrophoresis. The Colombia^5^-19 strain is from the Pneumococcal Molecular Epidemiology Network (www.sph.emory.edu/PMEN) (*17*).

During the epidemic, a subset of *S. pneumoniae* serotype 5 isolates (91 isolates), encompassing each year of the epidemic from the 4 affected western provinces were randomly selected and subjected to PFGE for restriction fragment-length polymorphism (RFLP) analysis (12 isolates in 2005, 26 in 2006, 13 in 2007, 20 in 2008, and 20 in 2009). All isolates typed by PFGE had either an identical RFLP pattern or differed by 1 band (Figure 4). Extending the RFLP analysis back to the 7 serotype 5 isolates from Canada from 2000 through 2004 showed that the first similar fingerprint detected was from a person who lived in a small town in rural southeastern Alberta in March 2004.

To determine whether this clone had been found in the United States, we compared it with 6 serotype 5 isolates from the US Centers for Disease Control and Prevention. Three isolates were from a small cluster of cases in San Francisco, California, in 2002 and 3 were from sporadic cases in the United States in 2006 (B. Beall, pers. comm.). Of these 6 isolates, the RFLP pattern for 5 isolates was identical to that of the epidemic clone and 1 isolate had a single band difference, suggesting that the serotype 5 clone in western Canada had been circulating in the United States in 2002 and 2006. This clone might have been imported into Canada from the United States; however, it might also have been imported from elsewhere in the world because sequence type (ST) 289 is the major circulating serotype 5 clone.

MLST analysis showed the allelic profile of the *S. pneumoniae* serotype 5 clone to be ST289 (*aroE16*, *gdh12*, *gki9*, *recP1*, *spi41*, *xpt33*, *ddl33*). ST289 has been listed in the MLST database (http://spneumoniae.mlst.net). The ST289 clone was originally reported from Colombia and is contained in the Pneumococcal Molecular Epidemiology Network list of worldwide antimicrobial drug–resistant clones (designation Colombia^5^-19) (www.sph.emory.edu/PMEN) (*17*).

## Discussion

Large epidemics of pneumococcal disease might go unrecognized unless surveillance programs are in place to document fluctuations in serotype prevalence, as reported here. The year-to-year variability of invasive pneumococcal disease caused by *S. pneumoniae* serotype 5 seen in some countries might actually reflect serotype 5 outbreaks similar to what we have described (*18*). For example, in 2000 in Mali, Africa, 50% of the isolates recovered from children with invasive pneumococcal disease were serotype 5, yet 2 years later; this percentage had dropped to a small portion of the total cases (*19**,**20*). This serotype 5 variability has also been reported in Chile and Israel (*21**,**22*). In Israel during 1989–1998, serotype 5 was the second most common serotype (serotype 1 was the most common) that caused invasive pneumococcal disease (12%–13% of cases among children <15 years of age) (*21*).

Although in other countries the number of *S. pneumoniae* serotype 5 cases might vary from year to year, in Canada no variability for serotype 5 was evident until the 2005–2009 epidemic. Few serotype 5 isolates had been documented since 1991, when the National Centre for Streptococcus first began performing pneumococcal serotyping to support national surveillance in Canada, until 2005. This serotype 5 strain has been demonstrated elsewhere in the world, not just Canada. Data from the MLST database and published reports indicate that the Colombia^5^ ST289 clone has been reported in countries in Europe, Latin America, and Africa and in the United States (http://spneumoniae.mlst.net) (*23**–**26*). In addition, the rate of resistance to trimethoprim/sulfamethoxazole by the Colombia^5^ ST289 clone has been reported as 80.8% (*27*) and 58.2% (*17*) of the Colombia^5^ ST289 strains from Latin American countries.

The *S. pneumoniae* serotype 5 epidemic mostly affected middle-aged men (median 41 years of age). Other risk factors were homelessness and First Nations heritage, although these factors accounted for a small percentage of the population. Because invasive pneumococcal disease reportedly affects homeless populations, the finding that homelessness was a major demographic factor associated with this epidemic is not surprising (*28**–**30*). A recent study from Toronto, Ontario, Canada, found that incidence of invasive pneumococcal disease was greater in the homeless population than in the general population (*30*) and that the variables associated with the serotype 5 epidemic (tobacco use, alcohol abuse, illicit drug use) were associated with invasive pneumococcal disease. Serotype 5 pneumococci were not identified in this study.

In December 2006, investigators found *S. pneumoniae* serotype 5 affecting persons of First Nations heritage living near the city of Calgary, Alberta, and persons living in inner-city Calgary; Edmonton; and Vancouver, British Columbia (*31**–**33*). These reports indicated that the variables associated with invasive pneumococcal disease caused by this serotype were homelessness, use of illicit drugs, First Nations heritage, alcoholism, and hepatitis B or C, thereby corroborating our findings for those cases in northern Alberta (*31**,**32*). Recovery of this serotype in locations other than inner cities in western Canada (including northern Saskatchewan) suggests its spread beyond the larger metropolitan areas of western Canada (*34*).

A public health response to the epidemic occurred throughout western Canada. Regional health authorities conducted vaccination programs focused primarily on homeless populations in large metropolitan areas. They used the 23-valent pneumococcal polysaccharide vaccine, which contains serotype 5. As a result of these large-scale pneumococcal vaccination campaigns, the National Advisory Committee on Immunization issued an advisory statement recommending use of the 23-valent pneumococcal vaccine for homeless persons and injection drug users (*35*). Examples of public health measures used to address the outbreak in 2 health regions are contained in reports from British Columbia focusing on *S. pneumoniae* serotype 5 outbreaks in the Vancouver downtown eastside and in the city of Kelowna (*35**,**36*). In Vancouver, investigators found that the serotype 5 strain accounted for 78% of cases of invasive pneumococcal disease. The major risk factors reported were use of crack cocaine and residence in Vancouver’s downtown eastside, an impoverished part of that city where most of the illicit-drug users and homeless persons live (*36*). As a result, Vancouver Coastal Health authorities targeted rooming houses, shelters, food banks, and other community locations (*32*). In Kelowna, public health nurses and health care providers focused a pneumococcal vaccination program on persons who were homeless and/or addicted to illicit drugs or alcohol; at the time of their report, they had vaccinated ≈1,000 at-risk persons (*37*).

A strength of our study is the ability of the centralized laboratory to capture and document shifts in the epidemiology of pneumococci in Canada. Regionalization of serotyping of pneumococci has the potential to miss changes in serotypes that can occur rapidly.

A weakness of our study is the lack of clinical data for all cases of invasive pneumococcal disease caused by *S. pneumoniae* serotype 5 that occurred during this epidemic. Logistically, gathering all of these data was not possible; however, the clinical data from northern Alberta do indicate some of the clinical variables involved and the concurrent conditions associated with serotype 5 cases. Another limitation might be that the variables for persons with invasive pneumococcal disease caused by *S. pneumoniae* serotype 5 (patient demographics, substance-abuse associations, concurrent conditions, type of pneumococcal disease, and outcomes) were compared with those for persons with other pneumococcal disease rather than with a healthy (nondiseased) control group. However, we thought it useful to try and determine among those with invasive pneumococcal disease whether differences existed among disease caused by serotype 5 and other serotypes.

We do not know why the epidemic was focused in western Canada and why large numbers of cases did not spread to eastern Canada or the United States. Clearly, we do not understand all the dynamics associated with large invasive pneumococcal disease epidemics.

In conclusion, we document a rare large-scale outbreak of invasive pneumococcal disease in western Canada caused by a single clone of *S. pneumoniae*. The clone possessed a serotype 5 polysaccharide capsule and ST289, indicating that the clone is derived from the international Pneumococcal Molecular Epidemiology Network clone Colombia^5^-19 originally described in Colombia (*18*). RFLP comparing a collection of *S. pneumoniae* serotype 5 isolates from the United States with the epidemic clone from western Canada showed that all isolates were identical, suggesting that this strain has been circulating within the United States. However, without direct evidence, we do not know from what part of the world this clone was originally imported into Canada.
